# Effects of Heat Treatment on Microstructures and Mechanical Properties of a Low-Alloy Cylinder Liner

**DOI:** 10.3390/ma17040802

**Published:** 2024-02-07

**Authors:** Wenjuan Zhang, Hao Gao, Dong Liu, Ying Gao, Yuqing Zhang, Lingchao Kong

**Affiliations:** 1 School of Mechanical and Electric Engineering, Sanming University, Sanming 365004, China; gaohaozunp@sohu.com; 2 ZYNP International Corporation, Industrial Cluster District, Mengzhou 454750, China; zynpliudong@163.com (D.L.); 15139103066@139.com (Y.G.); 3 School of Material Science and Engineering, Chongqing University, Chongqing 400044, China; zhangyuqing620@sina.com; 4 Fujian Taiming Casting Pipe Technology Co., Ltd., Sanming 365004, China; konglc0007@sina.com

**Keywords:** low-alloy cast bainite, heat treatment, hardness, tensile strength, wear resistance

## Abstract

Cylinder liners, considered a crucial component of internal combustion (IC) engines, often require excellent mechanical properties to ensure optimal engine performance under elevated temperatures, pressures, and varying loads. In this work, a new low-alloy cylinder liner, incorporating a low content of molybdenum, copper, and chromium into gray cast iron, was fabricated using a centrifugal casting process. Subsequently, the heat treatment processes were designed to achieve bainite microstructures in the cylinder liner through rapid air cooling, isothermal transformation, and tempering. The effects of different air-cooling rates and tempering temperatures on the microstructure evolution and mechanical properties of cylinder liner were investigated. The results revealed that during the supercooled austenite transformation process, rapid air cooling at a rate of 14.5–23.3 °C/s can effectively bypass the formation of pearlitic structures and directly induce the formation of bainite structures. Once the temperature exceeded 480–520 °C, hardness and tensile strength increased with the temperature increase owing to the enhancement of the lower bainite content, the reduction of residual austenite, and the precipitation of the fine hard carbides in the matrix. With temperatures above 520–550 °C, the carbide and lower bainite organization coarsened, thereby reducing the hardness and tensile strength of the material. Therefore, the optimal heat treatment parameters were rapid cooling at 14.5–23.3 °C/s rate to obtain bainite, and tempering of 480–520 °C for finer and more uniform bainite. In addition, the results of the characterization of the mechanical properties of the cylinder liner after heat treatment showed that the hardness, tensile strength, and wear resistance were improved with the refinement of the bainite.

## 1. Introduction

The internal combustion (IC) engine manufacturing industry plays a vital role in achieving green development and double-carbon targets [1]. Improving thermal efficiency is crucial to reducing carbon emissions from IC engines. However, a substantial increase in thermal efficiency can significantly increase the engine’s burst pressure, leading to serious deterioration of the lubrication environment. As a key component of the IC engine, the cylinder liner can be severely worn when it directly interacts with elevated temperatures, high-pressure gas, and the rapid movement of the piston. Therefore, excellent mechanical properties of cylinder liners are essential to withstand harsh environments, such as high hardness, exceptional tensile strength, and good wear resistance [2]. The cylinder liners are manufactured by centrifugal casting and subsequently undergo heat treatment. Heat treatment greatly affects the liners’ microstructures and mechanical properties; therefore, studying heat treatment parameters is decisive for developing high-performance cylinder liners and improving the reliability and service life of IC engines [3].

Gray-cast iron is widely used in the manufacturing of cylinder liners [4]. However, its properties do not meet the high-performance requirements of modern IC engines. Numerous studies have been reported on improving the performance indicators of gray cast iron. For instance, researchers have proposed using composite materials [5,6] and applying surface modification technologies [7,8,9,10,11,12] to enhance the mechanical properties and wear resistance of cylinder liners. However, cylinder liner materials such as boron alloy cast iron, high-phosphorus cast iron, and vanadium–titanium cast iron have higher wear rates [13], lower strength, and lesser wear resistance [14] due to the increased spacing between pearlite structures in cast iron. The tensile strength of these materials is low, with σ_b_ ranging from 200 MPa to 300 MPa and hardness from 210 HB to 260 HB. These values do not meet the high burst pressure requirements of modern IC engines. Therefore, the research and development of new cylinder liner materials are essential to meet high-standard emission requirements. In this perspective, as-cast bainite is a good substitute due to its high mechanical properties and wear resistance [15]. Substantial research has been conducted on obtaining bainite microstructures.

Adding various alloying elements can improve the mechanical properties of gray cast iron while satisfying the requirements of harsh working conditions [16,17,18,19]. For instance, Zhou et al. [20] found that adding copper helps obtain austenite and bainite microstructures through a three-step heat treatment process, improving the material’s yield and tensile strength. Shantanu and Kuldeep [21] studied the effects of molybdenum and copper in gray cast iron on mechanical properties. They found that by increasing the proportions of molybdenum and copper in gray cast iron, the material’s tensile strength and hardness increased, and impact energy decreased. Jose et al. [22] studied the effect of different nickel contents on bainite and found that nickel slowed down the transformation kinetics of pearlite and ferrite phases, favoring the formation of martensite and bainite. Numerous studies have shown that obtaining bainite through alloying improved the austempering process, thereby obtaining the bainite’s matrix microstructures and improving its mechanical properties. Krzysztof et al. [23] proposed a heat treatment of the medium carbon steel surface containing 20%, 40%, and 100% bainite transformation by combining bainitization and quenching partitioning processes. Their study showed that the quenching treatment can form multiphase microstructures comprising nano-bainite, ultrafine martensite, and retained austenite, thereby improving the material’s hardness and wear resistance. Bakhtiari and Ekrami [24] investigated high-bainitic dual-phase steel and obtained different bainite morphologies by changing the austempering temperature. They found that the samples’ yield strength, tensile strength, and hardness quenched at 450 °C increased due to the formation of finer bainite microstructures. Li et al. [25] proposed an austempering treatment process to obtain the mixed microstructures of bainite, martensite, and retained austenite. The evolution of bainite microstructures and its properties under different quenching parameters were studied. Their study revealed that the material’s tensile strength, toughness, and work hardening rate increased with the increased volume fraction of bainite. These studies imply that adding appropriate alloying elements and adjusting heat treatment parameters to obtain bainite microstructures can effectively improve the mechanical properties of cylinder liners. Sarkar et al. [26] used the salt bath isothermal quenching method for heat treatment of copper-alloyed gray cast iron to obtain bainitic cast iron microstructures. The results showed that the hardness and tensile strength of bainitic gray cast iron increased and decreased with the extension of isothermal hold time up to 60 min. With increasing isothermal holding temperature, the hardness and tensile strength of the alloy decreased. Hsu et al. [27] used isothermal quenching heat treatment to obtain bainite structures. It was shown that the hardness values of the heat-treated bainitic gray cast iron were increased compared to the as-cast microstructures. While the previous studies revealed the effect of isothermal quenching parameters on the bainite microstructures and mechanical properties, there remains a challenge to find a suitable method for industrial production of bainitic cylinder liners.

In this study, a new cylinder liner was manufactured by a centrifugal casting process in industrial conditions, incorporating low levels of molybdenum, copper, and chromium into gray cast iron. A heat treatment process was designed to obtain the bainite microstructures. The effects of air-cooling rate, insulation, and tempering on the microstructures of cylinder liners were studied. Experiments were conducted to evaluate liners’ hardness, tensile strength, and wear resistance under different heat treatment parameters. The optimal heat treatment parameters of the cylinder liner were determined through a comprehensive evaluation of their microstructures and performance, including a 14.5–23.3 °C/s air cooling rate and a 480–520 °C tempering temperature. This study provides a theoretical foundation for obtaining cylinder liners with bainite microstructures and excellent mechanical properties.

## 2. Materials and Methods

**Figure 1:**
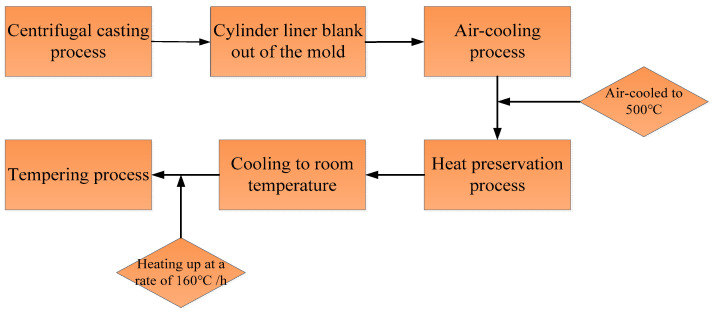
Fabrication process of the cylinder liners.

**Figure 2:**
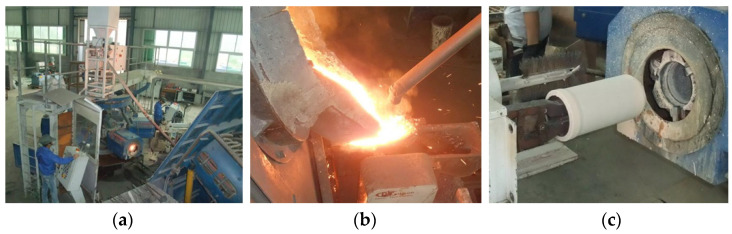
The centrifugal casting process of the cylinder liners: (**a**) centrifugal casting machine; (**b**) molten iron pouring out of the furnace; (**c**) demolding process of cylinder liners.

The chemical composition of cast iron studied in this work is presented in Table 1. The alloying elements of molybdenum, copper, and chromium were added into the cast iron to improve the properties of corrosion and wear resistance of cylinder liners. The fabrication process of cylinder liners includes the centrifugal casting process and subsequent heat treatment, as illustrated in Figure 1. Figure 2 exhibits the centrifugal casting process of cylinder liners. The material was melted in a 500 kg medium-frequency induction furnace, and subsequently shaped into cylinder liners using an automated horizontal centrifugal casting machine, the ZNJZ-8 (Jinan Intelligent Technology Ltd., Jinan, China). The ironing temperature ranged from 1480 °C to 1520 °C, the casting temperature ranged from 1420 °C to 1450 °C, and the out-mold temperature ranged from 850 °C to 890 °C. It is noted that the temperature of the billet after being removed from the mold depends on the cooling time in actual production [28,29]. Previous study has shown that the incoming water time has a great effect on the graphite morphology of cylinder liners, which often have A-type graphite, with fatigue resistance and fewer dendritic aggregation [30], as shown in Table 2. Therefore, experimental results of the effect of incoming water time of 15 s and 20 s, exchanging time of 25 s, 30 s, and 35 s, and reduction time of 30 s, 40 s, and 50 s on the mold-out temperature are shown in Table 3. To austenitize the casting and increase productivity, an incoming time of 15 s, exchanging time of 35 s, and a reduction time within the range of 30–50 s were employed. According to the fabrication process depicted in Figure 1, the cylinder liners, after being ejected from the mold, were rapidly cooled to 500 °C using compressed air, and then placed in a holding tank for heat preservation. The temperature of the heat preservation box was maintained within a temperature range of 320–400 °C for 90 min. Subsequently, the cylinder liner blanks were removed and cooled to room temperature.

**Figure 3:**
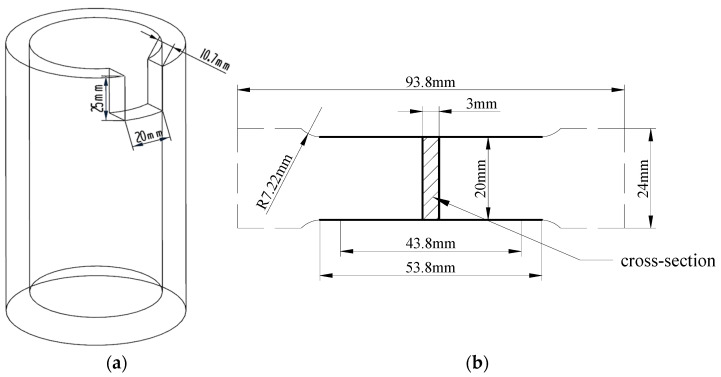
Schematic diagram depicting (**a**) the sampling location and size of specimens for microstructure observation, and (**b**) the specimens designated for tensile testing.

It is worth noting that the air-cooling rate, heat preservation process, and tempering temperatures have an important effect on the microstructures and properties of cylinder liners. In this work, eight cylinder liners were fabricated using a centrifugal casting process to investigate the influence of air-cooling rate on the microstructures and properties. The air-cooling rates were 11.6 °C/s, 12.9 °C/s, 14.5 °C/s, 16.6 °C/s, 19.4 °C/s, and 23.3 °C/s to test. Fourteen specimens with a size of 20 mm × 25 mm × 10.7 mm were separated from each cylinder liner, as illustrated in Figure 3a. These specimens were prepared for tempering treatment. Each specimen was individually heated at a rate of 160 °C/h to temperatures of 250 °C, 300 °C, 350 °C, 400 °C, 450 °C, 500 °C, and 550 °C, respectively. They were then held at each temperature for a duration of 2 h before being cooled down to 200 °C in the furnace and subsequently gradually cooled to room temperature.

**Figure 4:**
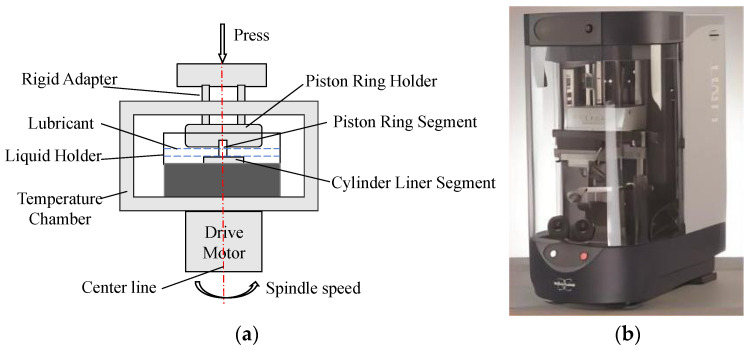
Schematic illustration of (**a**) wear tests and (**b**) the wear testing machine.

An optical metallographic microscope (Leica’s DM2500M, Wetzlar, Germany) and scanning electron microscope EVO18 (SEM with Tungsten Filament, Zeiss, Jena, Germany) were applied to explore the influence of air-cooling parameters, isothermal transformation, and tempering temperatures on the microstructures of the cylinder liners. Moreover, the microstructure composition of the cylinder liners was analyzed using Leica’s metallurgical microscope DM2500M with analysis software Profound iron&Steel, version 8.1 of the metallographic image analysis system. Tensile tests, hardness tests, and wear resistance tests were employed to investigate the influence of air-cooling parameters and tempering temperature on the properties of cylinder liners. Tensile tests were conducted at a room temperature using a WDW-300 KN testing machine (Wintime Machinery Co., Ltd., Guilin, China). The standard tensile specimens were machined according to GB/T228.1-2010 [31], as depicted in Figure 3b. Hardness tests were performed using a TH608 Brinell hardness tester (Beijing TIME High Technology Ltd., Beijing, China). Wear resistance tests were carried out on the UMT-3 wear testing machine (BRUKER, Billerica, MA, USA). A schematic illustration and the testing machine used in the wear tests are shown in Figure 4a,b, respectively. The heat-treated specimens were subjected to wear resistance tests under the following conditions: lubricant temperature of 50 °C, host speed of 350 r/min, and an applied load of 20 kg for 4 h. The wear resistance of cylinder liners was evaluated through the wear loss and wear morphologies on the surface of specimens.

## 3. Results and Analyses

### 3.1. Effect of Heat Treatment Parameters on the Microstructure of Cylinder Liners

#### 3.1.1. Characterization of Microstructures under Different Air-Cooling Parameters

**Figure 5:**
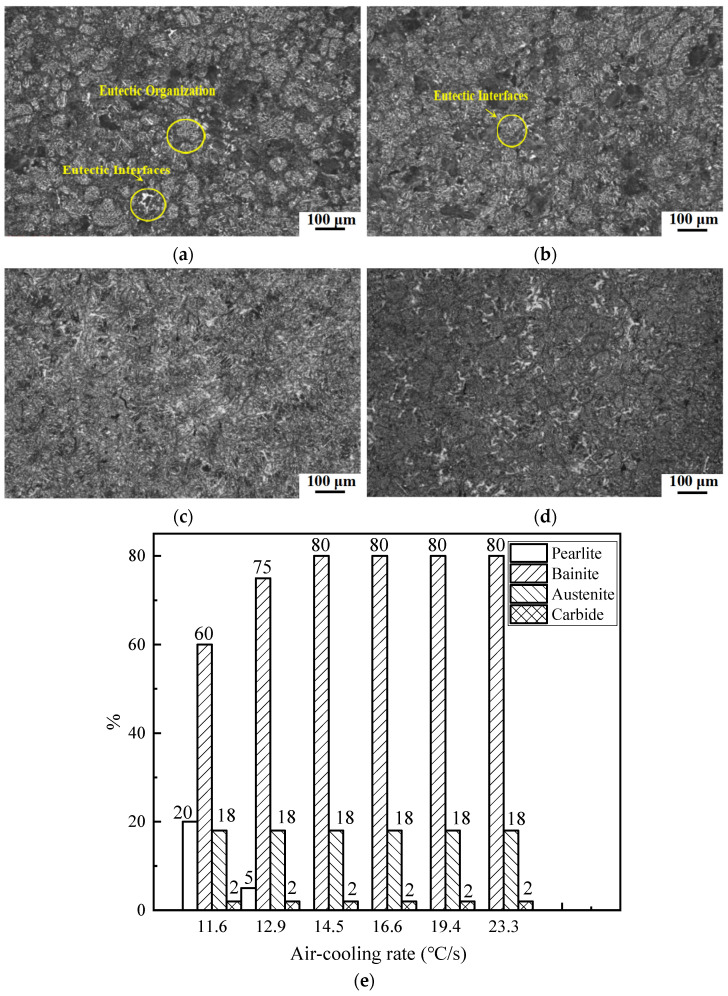
Optical microstructures of specimens subjected to air-cooling rates of (**a**) 11.6 °C/s, (**b**) 12.9 °C/s, (**c**) 14.5 °C/s, (**d**) 16.6 °C/s, and (**e**) the graph of variation of microstructure components with the cooling rate.

To analyze the effect of air-cooling parameters on the microstructures of cylinder liners, the specimens were rapidly cooled at different cooling rates and durations. Figure 5a–d illustrate the microstructures of cylinder liners under different air-cooling parameters. It can be seen from Figure 5a–d that during the cooling and solidification of the casting, eutectic cluster structures were formed, and interfaces were generated between these structures. With an increase in the cooling rate, the eutectic clusters were gradually refined and formed feathery bainite structures. At a cooling rate of 11.6 °C/s, the microstructure fraction was 20% pearlite (P), 60% bainite (B), 18% austenite (A), and 2% carbide. At 12.9 °C/s, the matrix organization comprised 5% P, 75% B, 18% A, and 2% carbide. When the cooling was 14.5 °C/s, 16.6 °C/s, 19.4 °C/s, and 23.3 °C/s, at corresponding reduced cooling times of 24 s, 21 s, 18 s, and 15 s, the microstructure fractions of cylinder liners in all cases were 80% B, 18% A, and 2% carbide, as depicted in Figure 5e. Table 4 presents the microstructure components corresponding to experimental air-cooling rates of 11.6 °C/s, 12.9 °C/s, 14.5 °C/s, 16.6 °C/s, 19.4 °C/s, and 23.3 °C/s, with corresponding cooling durations of 30 s, 27 s, 24 s, 21 s, 18 s, and 15 s, respectively, for a temperature difference of about 348 °C. The reason for this trend is that, after 14.5 °C/s, the cooling rate was increased, and the cooling duration was shortened, causing the supercooled austenite to bypass pearlite and undergo an as-cast bainite transformation, leading to the formation of a microstructure predominantly characterized by bainite, austenite, and carbide. 

#### 3.1.2. Characterization of Microstructures in Isothermal Transformation

**Figure 6:**
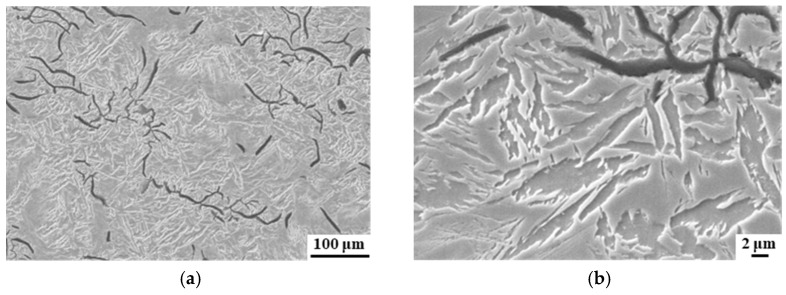
SEM microstructures showing the isothermal transformation of cast bainite at (**a**) low and (**b**) high magnifications.

The process of bainite isothermal transformation takes place in a heat preservation box. The blank was cooled as gradually as possible in the heat preservation box, while maintaining the temperature within the range of 320–400 °C, and holding it at this level for a duration of 90 min. As observed with scanning electron microscopy in Figure 6a,b, numerous bainite needles accumulated around the graphite sheet, with the untransformed carbon-rich austenite film and residual austenite occupying the vacant areas between graphite structures. The bainite needles grew from the surface of flake graphite particles, not as incomplete bainite needles, but rather as clusters of finer bainite subunits, exhibiting irregular distribution. This microstructure occurred when the cylinder liner entered the bainite transformation zone during the solidification process, and saturated ferrite nuclei precipitated onto the flake graphite. These nuclei caused the surrounding carbon-rich austenite to diffuse, which created a carbon-poor zone, ultimately leading to the formation of new bainite nuclei. This process resulted in a series of nuclei gradually growing into a bainite subunit structure, and repeated subunit growth led to the coarsening of the bainite needles. Since bainite nucleation and growth depend on graphite organization, only austenite remained in the spaces between the graphite particles. The presence of significant amounts of residual austenite and the accumulation of bainite near the graphite resulted in a coarse bainite structure with low hardness and strength, which do not meet the demanding requirements of high-performance engines and strict waste/gas emission standards. 

#### 3.1.3. Characterization of Microstructures in Tempering Process

**Figure 7:**
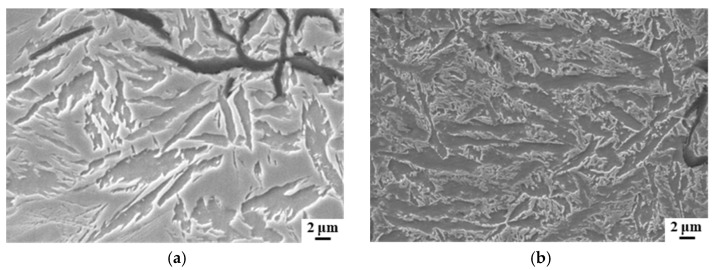
Comparison of SEM microstructures before and after tempering: (**a**) before tempering; (**b**) after tempering.

In the process of casting cylinder liners, stress is generated, particularly after the solid phase transformation and upon entering the elastic deformation region, due to temperature changes. This leads to inherent inhomogeneity and angular distortion within the structure. Furthermore, as depicted in Figure 6, the isothermal transformation process of bainite was characterized by a coarse tissue structure, requiring tempering of the specimens to achieve a uniform distribution of fine bainite structure. Figure 7 presents a comparison of the microstructures of specimens before and after tempering. It is seen that tempering resulted in a more uniformly distributed and finer needle-like bainite structure. The bainite needles were composed of smaller clusters of bainite subunits, with dispersed thin films of untransformed carbon-rich austenite. Post-tempering, recrystallization occurred in the original residual austenite and bainite organization. The presence of grain boundary defects facilitated the gradual precipitation of bainite nuclei, resulting in significant refinement of the needle-like bainite organization.

### 3.2. Effect of Heat Treatment Parameters on the Mechanical Properties of Cylinder Liners

#### 3.2.1. Effect of Air-Cooling Parameters on Hardness and Tensile Strength

**Figure 8:**
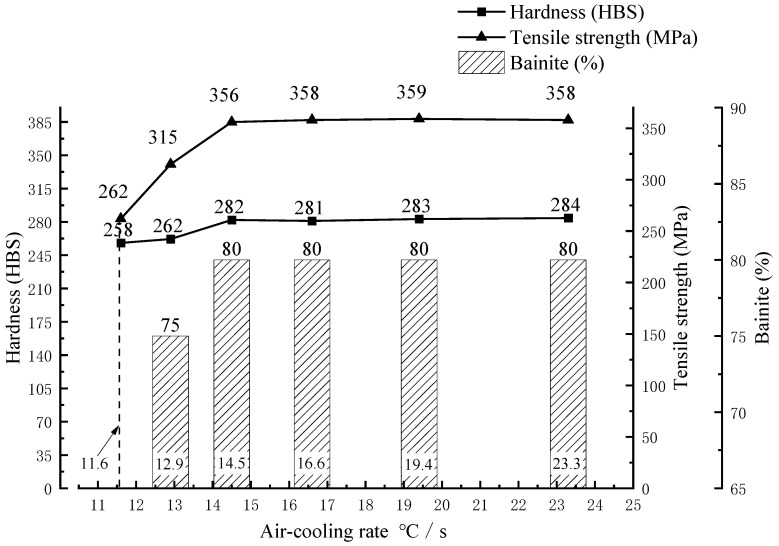
The variation in the average values of hardness, tensile strength, and the fraction of bainite in the specimens subjected to different air-cooling rates.

The variation in hardness and tensile strength of the specimens subjected to different air-cooling rates is depicted in Figure 8. At a cooling rate of 11.6 °C/s, the average values of hardness and tensile strength of the specimens were 258 HBS and 262 MPa, respectively, with no observed transformation of the bainite microstructures. When the cooling rate was increased to 12.9 °C/s, the average value of hardness increased to 262 HBS, and the tensile strength increased to 315 MPa, accompanied by a transformation of the bainite microstructures. The hardness and tensile strength of the castings continued to increase as the cooling rate was increased further. Specifically, the average value of hardness reached 282 HBS, and the tensile strength reached 356 MPa, accompanied by an increase in the transformation of the bainite microstructures, at an air cooling rate of 14.5 °C/s. Further transformation of the bainite microstructures occurred in the range of air-cooling rates of 14.5–23.3 °C/s. In this range of air-cooling rates, the average hardness of the specimens was 282.5 HBS, and the average tensile strength was 357.8 MPa. Compared with the stage with no bainite transformation, the hardness increased by 26 HBS, and the tensile strength increased by 97 MPa. The increase in hardness and tensile strength with a higher cooling rate is attributed to the realization of eutectic cluster refinement, the shear resistance at grain boundaries that inhibits bainite growth, which ultimately improves the stability of the supercooled austenite in bainite transformation [32] (see Figure 5c,d, for instance).

#### 3.2.2. Effect of Tempering Temperature on Hardness and Tensile Strength

Table 5 presents the average values of hardness and tensile strength for specimens tempered within the range of 250–550 °C. The cylinder liner specimens were subjected to tempering heat treatment at an air-cooling rate of 14.5 °C/s for 24 s and exhibited an initial average hardness of 281 HBS and tensile strength of 358 MPa before tempering. The tempering temperatures were incremented in intervals of 50 °C, and the hardness and tensile strength of the specimens were measured at each tempering temperature. The hardness and tensile strength of the cylinder liners remained relatively stable in the temperature range of 250–350 °C during tempering. However, at 400 °C, 450 °C, and 500 °C, there was a notable increase in the hardness and tensile strength of the specimens. However, both the hardness and the tensile strength decreased significantly at 550 °C.

**Figure 9:**
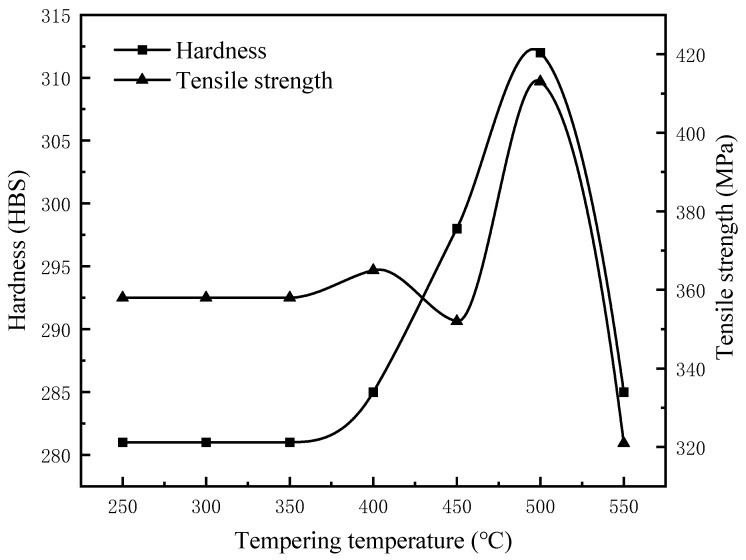
The variation in hardness and tensile strength at tempering temperatures in the range of 250–550 °C.

Figure 9 illustrates that within the tempering temperature range of 250–350 °C, there was no significant change in hardness and tensile strength compared to the pre-tempered state of the specimens. In the temperature range of 350–450 °C, although there was a slight increase in hardness, the magnitude of the increase was minimal, while the tensile strength decreased, indicating inadequate enhancement of the mechanical properties of cast bainite. Conversely, at temperatures in the range 450–530 °C, both hardness and tensile strength exhibited significant increases after tempering. However, it is important to note that within this temperature range, there is a relatively large span of temperatures. To better specify the range of tempering temperatures for improving the mechanical properties of cylinder liners, the temperatures were subdivided in the range of 470–550 °C. 

Table 6 presents the average values of hardness and tensile strength of the cylinder liner specimens at tempering temperatures ranging from 470 °C to 550 °C in 10 °C increments following subdivision. Between 480 °C and 520 °C, the hardness and tensile strength of the specimens significantly exceeded the pre-tempering levels of 281 HBS and 358 MPa, respectively. Then, the average hardness of cast bainite was 310 HBS and the average tensile strength was 384.8 MPa in the temperature range of 480–520 °C.

**Figure 10:**
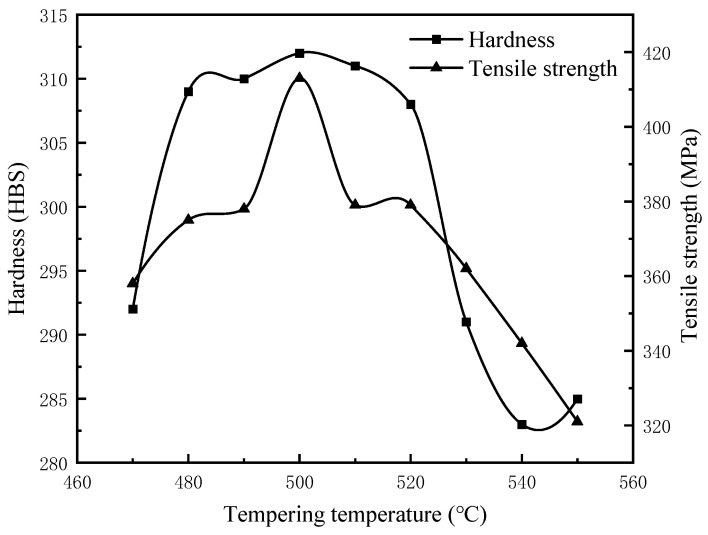
The variation in hardness and tensile strength in a tempering temperature range of 470–550 °C.

It was interesting to observe that the maximum hardness was 312 HBS and the maximum tensile strength was 413 MPa at 500 °C (e.g., the highest point in Figure 10), representing an increase of 31 HBS and 55 MPa of the hardness and tensile strength, respectively, from the pre-tempered state. By comparison with the results in reference [26], the hardness of 266 HBS and strength of 262 MPa of bainitic gray cast iron obtained at an isothermal-quenching 335 °C holding condition for 30 min were improved by 46 HBS and 151 MPa, correspondingly. This can be attributed to an increased proportion of lower bainite (e.g., Figure 7b) and the presence of hard diffuse carbides with increases in tempering temperature. The increased proportion of lower bainite and the presence of hard diffuse carbides led to adequate lower bainite transformation, reduced residual austenite content, precipitation of fine carbides in the matrix, and a consequent increase in the hardness and tensile strength of the casting. At temperatures in the range of 520–550 °C, carbide and lower-bainite structures coarsened, leading to decreased material hardness and tensile strength. In summary, maintaining the tempering temperature in the range of 480–520 °C resulted in improved mechanical properties of the specimens.

#### 3.2.3. Wear Testing of Heat-Treated Cylinder Liners 

To further evaluate the wear resistance of the cylinder liners following heat treatment, a test was conducted using a cylinder liner-piston ring fast wear tester to compare boron cast iron and low-alloy bainite. Boron cast iron is widely used in the manufacture of cylinder liners due to its exceptional wear resistance [33]. The tensile strength of boron cast iron cylinder liners ranges from 260 MPa to 310 MPa, and the hardness is 200–260 HBS [29]. To further evaluate the wear resistance of low-alloy bainite cylinder liners, the wear resistance of cylinder liners made of boron cast iron and low-alloy bainite was compared by analyzing wear loss and surface morphology, respectively. Both materials underwent the heat treatment process outlined in this study. Then, these two different cylinder liner materials were subjected to wear tests under the same conditions as previously mentioned. Following the conclusion of the tests, the average performance of the two types of cylinder liner materials was evaluated, as shown in Table 7. Wear loss in the X-X and Y-Y directions, which were perpendicular to each other, was separately measured at the cylinder liner bore. The X-X direction was parallel to the engine crankshaft, while the Y-Y direction was perpendicular to it, with the cylinder liner assembly serving as the reference for the engine position. The total average wear loss for the two materials was calculated. The boron cast iron cylinder liner had a total average wear loss of 0.044 mm, while the low-alloy cast bainite exhibited a total average wear loss of 0.014 mm. This signifies a reduction in wear loss of 0.03 mm.

**Figure 11:**
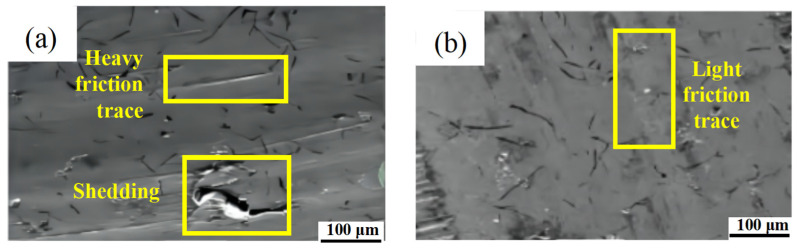
Surface wear morphologies of boron cast iron and low-alloy cast bainite cylinder liners: (**a**) boron cast iron cylinder liner; (**b**) low-alloy cast bainite cylinder liner.

Figure 11 shows surface morphologies of the boron cast iron and low-alloy cast bainite cylinder liners after the fast grinding test. The figure illustrates that the boron cast iron cylinder liner exhibits significant surface wear, with noticeable wear marks and small pieces falling off. Adhesive wear is also evident (see Figure 11a). In contrast, the low-alloy cast bainite cylinder sleeve has a relatively smooth and flat surface, with lighter wear marks on the base body (see Figure 11b). Through analysis of the cylinder liner’s wear surface, it was observed that the boron cast iron matrix cylinder liner generated wear debris during the wear process. This debris underwent extrusion, forming abrasive particles with higher hardness. These particles continuously exerted pressure on the friction pair’s surface, leading to the occurrence of fatigue cracks and local shedding.

Cast bainite cylinder liners exhibit enhanced strength and wear resistance due to the abundance of dislocations and the dislocation substructure present in the retained austenite. The high density of dislocations restricted their movement, thereby enhancing the strength and wear resistance of cast bainite. Additionally, a solid solution strengthening effect occurred as the cast bainite underwent a phase transformation process, leading to the diffusion of carbon atoms. This resulted in the presence of oversaturated carbon in the ferrite matrix, causing distortion resulting in a non-cubic-type crystal structure. The presence of carbon also increased the strength of the bainite organization through solid solution strengthening.

## 4. Conclusions

This work fabricated a new low-alloy cylinder liner and investigated the heat treatment parameters on the microstructures and mechanical properties of the fabricated cylinder liner. The results show that the microstructures of the low-alloy cylinder liner were characterized as bainite, austenite, and carbides after centrifugal casting, air-cooling, isothermal bainite transformation, and tempering. The heat treatment parameters were carefully controlled to bypass the formation of pearlite and promote the development of finer and homogeneous bainite. To obtain the desired microstructure characteristics and enhanced mechanical properties, the air-cooling rate was optimized within the range of 14.5–23.3 °C/s, and the tempering temperature was controlled within the range of 480–520 °C. The resulting specimens of the cylinder liner exhibited tensile strength of 413 MPa and average hardnesses of 312 HBS. In addition, the low-alloy bainite cylinder liner showed greater wear resistance when compared to the boron cast iron liner. This work provides a cost-effective alternative to conventional cylinder liners and supports the requirements of practical industrial production applications.

## Figures and Tables

**Table 1:** Chemical composition (wt%) of the cylinder liners.

C	Mo	Cu	Si	Mn	Cr	P	S
2.95	0.55	0.79	2.39	0.51	0.20	0.040	0.045

**Table 2:** Effect of incoming water time on the graphite morphology of the cylinder liners.

Incoming Water Time (s)	Metallographic Morphology		
	Graphite Types	Dendrite	Aggregation
5	E	yes	yes
10	D + A	yes	yes
15	50% A	no	no
20	50% A	no	no

**Table 3:** The mold-out temperatures correspond to various incoming water times, exchanging times, and reduction times.

Incoming Water Time (s)	Exchanging Time (s)	Reduction Time (s)	Mold-Out Temperature (°C)
15	25	30	932
		40	925
		50	911
	30	30	910
		40	899
		50	886
	35	30	890
		40	876
		50	868
20	25	30	912
		40	895
		50	882
	30	30	892
		40	879
		50	865
	35	30	876
		40	865
		50	855

**Table 4:** Microstructures obtained under different air-cooling parameters.

No.	Cooling Speeds (°C/s)	Cooling Times (s)	Microstructures
1	11.6	30	20% P + 60% B + 18% A + 2% Carbide
2	12.9	27	5% P + 75% B + 18% A + 2% Carbide
3	14.5	24	80% B + 18% A + 2% Carbide
4	16.6	21	80% B + 18% A + 2% Carbide
5	19.4	18	80% B + 18% A + 2% Carbide
6	23.3	15	80% B + 18% A + 2% Carbide

**Table 5:** Mechanical properties at tempering temperatures in the range of 250–550 °C.

Tempering Temperature (°C)	After Tempering	
	Average Hardness (HBS)	Tensile Strength (MPa)
250	281	358
300	281	358
350	281	358
400	285	365
450	298	352
500	312	413
550	285	321

**Table 6:** Mechanical properties at a tempering temperature range of 470–550 °C.

Tempering Temperature (°C)	After Tempering	
	Average Hardness (HBS)	Tensile Strength (MPa)
470	292	358
480	309	375
490	310	378
500	312	413
510	311	379
520	308	379
530	291	362
540	283	342
550	285	321

**Table 7:** Wear loss for boron cast iron and low-alloy bainite cylinder liners (mm).

Wear Loss	Boron Cast Iron		Low-Alloy Bainite	
X-X	0.022	0.022	0.023	0.005
Y-Y	0.058	0.074	0.021	0.007
Average Value	0.040	0.048	0.022	0.006
Overall Averages	0.044		0.014	

X-X values measured in the parallel crankshaft direction; Y-Y values measured in the vertical crankshaft direction; Overall Averages = Mean of two groups/2.

## Data Availability

Data are contained within the article.
